# A century of anti-G straining maneuvers. Are there further changes in AGSM physical training that can improve +Gz tolerance? A scoping review

**DOI:** 10.3389/fphys.2026.1878591

**Published:** 2026-07-06

**Authors:** David G. Behm, Ethan D Lawson, Blake C. W. Martin

**Affiliations:** 1 School of Human Kinetics and Recreation, Memorial University of Newfoundland, Newfoundland and Labrador, St. John’s, NL, Canada; 2 Defence Research and Development Canada, Toronto Research Centrea, Toronto, ON, Canada

**Keywords:** +Gz forces, apnea, breath hold training, G-LOC, hook breathing, muscle endurance, valsalva

## Abstract

High +Gz force tolerance is vital for pilot survival and aircraft performance capabilities. Over the last century, devices such as anti-G suits, pressurized breathing, seat configurations, audio-visual displays to monitor physiological responses, and other techniques have been developed. Anti-G straining maneuvers (AGSM) were also developed to combat the cerebral blood flow reductions that could lead to vision loss, +Gz-force induced loss of consciousness (G-LOC), and pilot performance impairments. Centrifuge conditioning is a very effective task-specific +Gz tolerance training but with few facilities to support it. The recovery breath during AGSM’s “hook” breathing can compromise elevated arterial pressures needed to maintain consciousness. Hence it might be fruitful to use breath hold training techniques to prolong the Valsalva maneuver and decrease recovery breath frequency. In addition, the lower body and trunk isometric contractions force output during AGSM can diminish due to fatigue effects. There are contradictions in the literature regarding the effectiveness of muscle endurance and strength training as well as aerobic versus anaerobic training. Very little information is published regarding integrated physical and cognitive training as operating an aircraft does not just involve physiological processes. Is it possible after many decades of AGSM research to make meaningful improvements to pilot +Gz tolerance training? The objective of this scoping review is to examine the AGSM literature and find AGSM components that may allow for advancements that either in isolation or in combination could enhance +Gz tolerance, preventing or delaying visual impairments, performance deficits, and G-LOC.

## Introduction

The next generation of fighter jets present ever-increasing gravity (+Gz) tolerance challenges to pilots, as these jets tolerate much higher Gz forces than humans can currently tolerate. Maximum operational +Gz-force capability of the next generation of military jets remains at about 9G, limited by the pilot and not the airplane. While the maximum jet capabilities remain confidential, further technologies, training tactics, and strategies might enhance the pilot potential Gz tolerance. Hence, the full capacity of the aircraft cannot be fully leveraged due to physiological limitations. This is not a new phenomenon. Defence departments and researchers have been tackling this issue since soon after the World Wars (Burton, 1991a). The first review was published in 1938 (Armstrong and J.W., 1938) and the first muscular straining maneuver to enhance +Gz tolerance was proposed in 1933 in the UK by Steinforth (Vashisth et al., 2017). Anti-G straining maneuvers (AGSM) were developed and have been refined over approximately the last 75 years (post-1950s) (Buick, 1992; Burton, 1998; Burton, 2000a; Burton, 2000b). AGSM involves the implementation of intermittent Valsalva maneuvers with brief recovery breaths (hook breathing) while also performing lower body and trunk (core) isometric muscle contractions (Lyons et al., 1997; Convertino et al., 2003; DeAsis-Fernandez et al., 2025). The objective of AGSM is to prevent or delay the reduction of cerebral blood flow and hence maintain vision, consciousness, and performance capabilities (Lyons et al., 1997; Chen et al., 2004; Pires Junior et al., 2024).

High +Gz forces can also present serious health risks or complications. Research involving swine has demonstrated coronary ischaemia at high +Gz forces (i.e., +7 +Gz) (Laughlin et al., 1978), and ventricular hemorrhaging (Burton and MacKenzie, 1975; Burton and MacKenzie, 1976), while in humans: ateclectasis (lung collapse), altered ventilation/perfusion gradients (Fong and Fan, 1997), cardiac arrythmia (Leverett and Burton, 1979), conduction and rhythm disturbances, right bundle branch block and high-+Gz bradycardia (Whinnery and Whinnery, 1990), electrocardiography (ECG) dysrhythmias (Whinnery, 1990), and ventricular ectopy (Whinnery et al., 1990) are reported. Furthermore, in rodent models, repeated exposure to either short-duration +25 +Gz or sustained +10 +Gz acceleration leading to G-LOC can produce cytotoxic brain edema through ischemia-induced metabolic changes and lactate accumulation (Shahed et al., 1994). Based on the variety of possible cardiac contraindications, ECG monitoring might be considered to ensure optimum medical safety during high-Gz centrifuge training (Whinnery, 1990). However, earlier accounts indicate reluctance among some aircrew to accept ECG monitoring during centrifuge exposure, owing to concerns about potential detection of disqualifying cardiac dysrhythmias; the extent to which this remains applicable under current monitoring practices is unclear (Whinnery, 1990).

Even moderate +Gz forces (1-4 +Gz) can impair the ability to discriminate varying masses or loads adversely affecting afferent sensitivity and motor control (Darwood et al., 1991). A centrifuge profile up to 12 +Gz impaired aircraft-specific psychomotor performance (e.g., entering codes, matching aircraft altitude and speed on a display) up to 60 seconds post-G-LOC (Forster and Cammarota, 1993), while 3 +Gz loads impaired isometric joystick force matching, which may be attributed to less appropriate central commands (Girgenrath et al., 2005). Contradictory findings have reported that motor practice under 3 +Gz load can improve isometric force matching sensitivity (Gobel et al., 2006), whereas Guadiera et al (Guardiera et al., 2007) countered that 3 +Gz-induced problems with matching forces were not improved by frequent 3 +Gz centrifuge exposures. The accuracy of tracking and flight path movements can also be impaired at 3 +Gz, with the authors suggesting vestibulo-spinal disturbances (Guardiera et al., 2008; Guardiera et al., 2010). However, the same lab also indicated that 3 +Gz loads can affect force production but have no appreciable effects on flight performance and cognitive load of experienced pilots (Dalecki et al., 2010). A further difficulty with sustained AGSM is the possibility of time (duration) misperception (Jamin et al., 2004) interfering with the proper timing of recovery breaths and cockpit tasks. Thus, high +Gz forces not only affect flight performance but also vision loss and incapacitation.

While technology has also improved +Gz tolerance with the development of pressurized inflatable anti-G suits (Buick, 1992; Burton, 1992; Burton, 2000a; Burton, 2000b; Burns et al., 2001), modified or reclined seating positions (Burton, 1998; Burns et al., 2001), headrest geometry (Burns and Whinnery, 1984; Newman, 1997), and positive pressure breathing (Shaffstall and Burton, 1979; Burton, 1986; Buick, 1992; Pecaric and Buick, 1992; Clere et al., 1993), the pilots’ self-directed physical capacity to maintain cerebral blood flow is still a crucial component to preventing vision loss (i.e., loss of peripheral vision: tunnel vision to total loss), and G-LOC (Burton, 1991b; Buick, 1992; MacDougall et al., 1993; Slungaard et al., 2017). The brain is very sensitive to cellular hypoxia. With increasing +Gz loads, the sympathetic nervous system does not adequately respond to cerebral hypoxia. Cerebral hypoxia reserve time is typically 3–6 minutes, whereas physiological responses to elevated blood pressure are 6–9 seconds. Since intraocular pressure is typically higher than intracranial pressure, vision deficits are more sensitive to decreased perfusion and thus typically precede G-LOC. The mean duration of G-LOC has been reported at about 11.9 seconds (range 2 to 38 s) subsequently followed by cognitive incapacitation (confusion or disorientation) that can persist for approximately 16 seconds (range 2 to 97 s). During G-LOC, pilot incapacitation results in an inability to control the aircraft that can lead to loss of the aircraft and pilot (Akparibo et al., 2026). Common psychological reactions following G-LOC include confusion, disorientation, denial of G-LOC recognition, unreliability, altered judgment, embarrassment, dissociation, euphoria, anxiety, fear, antagonism, and a “give-up” attitude (Whinnery and Jones, 1987). Pilots denial (psychological suppression) of G-LOC is an important problem in reporting surveys and flying safety (Whinnery, 1988).

One might ask after these many decades of AGSM research whether it is possible to make meaningful improvements to pilot Gz tolerance training. Although it is unlikely that large magnitude alterations can be developed, even small improvements may save lives and aircraft. A survey of UK Royal Air Force aircrew reported that 14.8% experienced at least one episode of G-LOC and 32.2% reported at least one episode of almost loss of consciousness (A-LOC) (Slungaard et al., 2017). This G-LOC incidence was lower than a survey 12 years earlier (20.1%), attributable to the introduction of centrifuge training. Centrifuge training can induce baroreflex adaptations with experienced pilots demonstrating greater sensitivity and effective blood pressure control (Newman et al., 1998). Slungaard et al. (2017) also suggested that greater reductions in G-LOC incidence may be achieved with a combination of centrifuge training, correct execution of the AGSM, and specific strength training programs. In addition, based on breath hold (apnea) training, it may be possible to decrease the frequency of recovery breaths during hook breathing to decrease blood pressure fluctuations. Perhaps concurrent physical and cognitive training will allow for improved aerial performance control while also delaying the deleterious effects of fatigue (e.g., respiratory, leg, and trunk muscles).

The objective of this scoping review is to review the AGSM literature and find AGSM components that may allow for improvements (i.e., quite likely of small but meaningful and significant magnitude) that either in isolation or in combination could ameliorate +Gz tolerance and prevent or delay visual deficits, performance deficits, A-LOC and G-LOC. This may involve adapting ideas from other related athletic activities such as breath hold (apnea) or hypoventilation training techniques, respiratory fatigue training, strength, endurance, anaerobic (e.g., sprint training) or aerobic training, combined physical and cognitive training among others. Based on this review, new research avenues may be inspired to develop or modify new or established types of training or techniques.

## Centrifuge (high +Gz acceleration) conditioning

Centrifuges have a nearly 200-year history of use in human research and medical treatment (Meeker, 1991), with adoption for pilot training emerging during the World Wars (Convertino, 1998). As might be expected, based on the training specificity concept (Sale and MacDougall, 1981; Behm and Sale, 1993), centrifuge exposure will improve one’s tolerance to the stresses imposed by high +Gz forces with a centrifuge (Convertino, 1998). For example, a survey of centrifuge training indicated that G-LOC incapacitation may be reduced by 17 seconds and shorten the recovery by 9 seconds (Whinnery and Burton, 1987).

### Acute centrifuge exposure effects

Just experiencing a single +Gz exposure can increase resting blood pressure and total peripheral resistance indicating acute modifications in the cardiovascular system enhancing +Gz tolerance with subsequent exposures of less than 2 min (Stevenson et al., 2014). Simple response times increased with longer durations of 4.5 +Gz (Truszczynski et al., 2013). In a report to Defence Research and Development Canada, Buick (2004) further reported that a) relaxed +Gz tolerance increases with repeated 20 sec +Gz exposures relative to the initial exposure, b) tolerance is greater when repeated exposures are separated by 1-s rather than 15-s inter-cycle intervals, and c) peripheral vascular resistance increases after the first +Gz exposure, likely contributing to the observed increase in +Gz tolerance. In the period surrounding centrifuge training, tension arousal is elevated in advance of exposure (up to 2 h prior) and increases further immediately before centrifugation, while energetic arousal rises shortly before the run and remains elevated following exposure (Biernacki et al., 2012). Coping styles also modified the level of central nervous system arousal when exposed to +Gz stress (Biernacki et al., 2017). Whereas a single +Gz exposure establishes baseline tolerance, successive +Gz exposures are associated with further increases in +Gz tolerance, attributed to carryover compensatory mechanisms, primarily vasoconstriction, with possible contributions from enhanced venous return and baroreflex function (Lalande and Buick, 2009). These adaptations are reported to be rapid, occurring with only a few exposures. According to Vashisth et al. (2017) at least one centrifuge (high +Gz) session per week is needed to maintain maximum +Gz tolerance.

### Chronic or repeated centrifuge exposure effects

In rats, repeated moderate +Gz exposure (4 and 6 +Gz) induced significant increases in the intensity and duration of heat shock protein-70 mRNA activity compared to either low (2 +Gz) or high (10 +Gz) exposures. There was evidence of mild neuronal damage observed after 3–5 exposures at 4 +Gz, contrasting with significant hippocampal and cortical neuronal damage with repeated bouts at 10 +Gz (Jin-Sheng et al., 2002). Fortunately, Laughlin (1982) reported an absence of any cardiac damage in individuals who had many +Gz exposures over a 2-year period. As with exercise, moderate levels of stress can lead to protective adaptations, whereas high intensity activity or in this case, high +Gz levels, can induce damage.

Repeated exposures to high +Gz centrifuge conditioning can induce greater +Gz tolerance-related adaptations to blood pressure and cerebral perfusion in humans (Convertino, 1998). G-LOC occurrence is reported to be higher in pilots with less aircraft-specific flight time (Lyons et al., 1997; Green and Ford, 2006; Yun et al., 2019) and less experienced students (Sevilla and Gardner, 2005). Five weeks of centrifuge conditioning without performing AGSM or leg muscle contraction increased tolerance to rapid but not gradual +Gz increases, suggesting no significant change to the vascular sympathetic or carotid baroreflexes but reduced, vestibulosympathetic reflex responses (Eiken et al., 2025). In a previous study, Eiken et al. (2022) reported that five weeks of centrifuge training resulted in a 13% increase in relaxed rapid onset +Gz tolerance, but no corresponding improvement in gradual onset +Gz tolerance. This adaptation was accompanied by increased pressure resistance in dependent leg arteries and arterioles pressure resistance, but not the arms. In addition, training attenuated the initial decrease in arterial pressure during rapid-onset high G, but no change during gradual-onset +Gz loading. Collectively, these findings suggest that centrifuge-based +Gz conditioning primarily induces increased pressure resistance in dependent arteries/arterioles due to +Gz-induced high intravascular hydrostatic pressure gradients (Eiken et al., 2022). A review by Convertino (1998) reported that repeated exposure to high, sustained +Gz acceleration is associated with reduced orthostatic hypotension; venous compliance; lower-limb blood pooling; increased carotid-cardiac baroreflex sensitivity; attenuated stroke volume and cardiac output during orthostatism; increased α1-adrenoreceptor responsiveness; reduced vasoconstriction under lower-body negative pressure; and increased total blood and red blood cell volume. Repeated +Gz load also significantly improved post-exposure attention switching, however visuospatial memory reproduction deteriorated (Biernacki et al., 2013). Success in centrifuge training was positively associated with age (experience) and self-reported AGSM proficiency, but negatively associated with depression level (Yun et al., 2019). Centrifuge training is viewed as positive (73% enthusiastic) compared to only 2% of the 382 United States Air Force fighter pilots perceiving it to be negative (Gillingham and Fosdick, 1988).

## Non-physiological techniques to sustain high +Gz forces

Air combat maneuvering (ACM) by Australian air force F/A 18 pilots involves repetitive excursions above 7 +Gz (Newman and Callister, 1999). According to the US air force (USAF) the “informal” G-tolerance standards include the ability to sustain 7 +Gz for 15 seconds without total peripheral vision loss, or G-LOC while wearing an anti-G suit (Gillingham, 1987). These operational demands necessitate the use of non-physiological countermeasures to sustain performance and consciousness during high +Gz exposure.

Anti-G suits constitute the primary non-physiological countermeasure against +Gz stress. The anti-G suit contributes approximately 3.0 +Gz to total protection, and when combined with a pressure breathing system, can provide ≥ 4.6 +Gz protection overall (Eiken et al., 2007). Anti-G suits also reduce the duration of incapacitation from G-LOC by approximately 2 seconds allowing wearers to regain environmental awareness more rapidly (Forster et al., 1994). The absence of anti-G suits can have significant physiological consequences: without them, renal blood flow can be severely depressed leading to proteinuria during +Gz loads between 3.5 and 5.5 G. applied for 15 min (Noddeland et al., 1986).

When combined with anti-G suits, appropriate positive pressure breathing may provide protection up to 9 +Gz with minimal or no straining, while higher tolerances (up to 12 +Gz), have been achieved with the incorporation of additional design factors (Burns, 1988; Lu et al., 2005). A proposed schedule for adequate +Gz protection would be the application of 42 mm Hg pressure breathing at the start of +Gz forces increasing to a maximum pressure of at least 73 mm Hg. at 3.3 +Gz (Pecaric and Buick, 1992). Assisted positive pressure breathing (50 and 70 mmHg) has increased +Gz tolerance time (Burns and Balldin, 1988). While the pressure schedules recommended by Burns and Balladin have not been prescriptively adopted, positive pressure breathing has become a standardized component of air-crew life support systems, with NATO aeromedical publications specifying its integration with overall G-protection systems used by high performance fighters (Office NS, 2018).

The effectiveness of non-physiological systems can depend on their interaction with physiological responses. Tong et al. (1994) reported head and body position can influence acceleration tolerance; however, the anti-G-suit combined with the AGSM were found to overcome these positional effects.

Eiken et al. (2007) reported that pressure breathing is not additive with the protection provided by AGSM. Eiken et al. (2003) in another study indicated that arterial pressure was only 30–50 mm Hg higher with pressure breathing, suggesting pressure breathing makes only a minute contribution when combined with AGSM, as Valsalva maneuvers also increase arterial pressure by a similar extent (Porth et al., 1984). Arterial pressure increases of 30–50 mm Hg are not substantial when left ventricular pressure may reach 300 mm Hg with isometric resistance exercise (Laughlin, 1986).

Aircraft design features, in particular seat geometry, further influence achievable +Gz tolerance. Tilt-back seat angles greater than 55° in combination with AGSM and G suits have been associated with tolerance of up to 12 +Gz (Burns, 1988; Lu et al., 2005), highlighting the role of body orientation in reducing hydrostatic gradients during acceleration.

A mathematical model for predicting maximum G-level tolerances was provided by Burton (1998), incorporating both anti-G suit pressure and body reclination angle. Based on this model, predicted tolerances were as follows: 55° = 11.7 +Gz and 12.7 +Gz with 7 and 10 psi respectively; 65° = 12.1 and 12.8 +Gz with 6 and 9 psi; and 75° = 13.4 and 14.2 +Gz with 5 and 8 psi anti-G suit pressure respectively. These estimates illustrated the combined influence of suit pressure and posture on maximum +Gz tolerance.

Additional non-physiological strategies have been proposed to improve pilot performance under +Gz stress. Improvements in mask regulators have been recommended after findings that 23-33% of the inhalator mask cavity pressures exceeded the Air Standardization and Coordination Committee limits (Whitley, 1997). Nelson et al. (2001) reported that localization of virtual sound sources is well maintained at varying acceleration levels and suggesting spatial audio displays integrated into the human-computer interface could aid pilot performance at higher +Gz.

In summary, non-physiological anti-Gz technologies including centrifuge exposures with varying onset rates and durations, pressurized anti-G suits to increase lower body vascular resistance, pressure breathing with elevated oxygen concentrations, and tilt–back seat angles greater than 55°can substantially augment +Gz tolerance and permissible maximum +Gz loads. In addition to these external technologies, pilots can use specific techniques to further augment their own anti-+Gz physiological responses.

## Anti-G straining maneuver

AGSM comprises trunk and lower body isometric muscular contractions and a respiration maneuver known as hook breathing (MacDougall et al., 1993; Yang et al., 2021). The literature seems to be in agreement that AGSM is the most effective technique to prevent G-LOC (Pires Junior et al., 2024). The trunk and lower body isometric muscle contractions are an attempt to prevent blood pooling in the lower extremities. Higher +Gz tolerance is related to the ability to maintain sufficient levels of arterial pressure during +Gz load to sustain cerebral blood flow (Sundblad et al., 2016). Elevated arterial pressure with AGSM has been attributed to lower body muscle contraction, and this component is less fatiguing than the respiratory straining component (MacDougall et al., 1993). At 1 +Gz, pulsing leg isometric contractions were not more effective in elevating blood pressure than a constant isometric contraction (MacDougall et al., 1993). Voluntary muscle activity also increases muscle spindle afferent activity to the reticular activating system (Bagshaw and Whinnery, 1989). The reticular activating system regulates consciousness, wakefulness, and arousal (Parvizi and Damasio, 2001), which could play an important role in combatting G-LOC.

Simultaneously, hook breathing involves a preparatory inspiration of less than 1 s followed by a Valsalva maneuver (forceful exhalation against a closed glottis) for 3–4 s to elevate intrathoracic pressure and systemic arterial blood pressure at the aortic valve reducing blood pressure loss at the cranial level (Burton et al., 1974). The objective of constantly tensing muscles and cyclically repeating the Valsalva maneuver is to sustain adequate cerebral blood perfusion to withstand the sustained high +Gz forces. Arterial baroreceptor loading induced by repeated straining maneuvers increases aortic, but not carotid, cardiac baroreflex responses (MacDougall et al., 1993). Following repeated straining maneuvers, aortic cardiac baroreflex responsiveness remains increased for as long as three hours, which may provide pilots with protection from development of orthostatic hypotension (Convertino et al., 2003). In conjunction with isometric contractions, breath-hold enhances exercise-induced increases in mean arterial pressure as well as blood velocity in the middle and posterior cerebral arteries (Watanabe et al., 2022). Higher relaxed +Gz tolerance is associated with the capacity for maintained reflexive vasoconstriction and response of the vascular baroreflex during this orthostatic stress (Sundblad et al., 2016). Individuals with higher +Gz tolerance primarily sustain mean arterial pressure through increases in total peripheral resistance, whilst low +Gz tolerance individuals tend to demonstrate a greater dependency on reflex increases in cardiac output. Hence, vasoconstrictor reserve capacity (not cardiac output) demonstrates a relationship between +Gz tolerance and leg arterial distensibility (Sundblad et al., 2014). Precapillary leg vessel wall stiffness was found to be higher in individuals with high relaxed +Gz tolerance (Eiken et al., 2012). Thus, higher +Gz tolerance is associated with the ability to increase arterial resistance providing a greater vasoconstrictor reserve capacity.

Properly executed AGSM can increase tolerance by 4 +Gz, whereas poorly executed AGSM is the most commonly known factor for G-LOC (Whinnery, 1986; Lyons et al., 1997; Sevilla and Gardner, 2005; Tu et al., 2020a). Lyons et al. (1997) surveyed 78 USAF fast jet pilots who reported one or more problems with their AGSM (73%) such as the pacing of their breathing was too quick (42%: < 2 s cycle), or too slow (14%: > 4 s), inhalation was too long, and some pilots frequently or occasionally talked during +Gz exposures (44%).

Tu et al. (2020b) indicated that older age and lower BMI are correlated with decreased high-+Gz tolerance. However, a retrospective study by Forster et al. (1999), did not detect significant effects of age (20–61 years) on gradual and rapid onset +Gz tolerance in a centrifuge.

### Apnea/breath hold/valsalva physiological responses

Hook breathing cycles are recommended at 3-second intervals (Massaferri et al., 2025) to maintain sufficient blood pressure, although there is very little information in the literature on this relationship. According to the few reports (and personal experience), it is most challenging to maintain hypertension during the recovery breath (Lyons et al., 1997). Tunnel vision (progressive loss of peripheral vision), greyout (temporary dimming of vision), and G-LOC typically occur either during or immediately following the recovery breath when arterial pressure transiently declines, and is difficult to re-establish (Lyons et al., 1997).

Consequently, the authors hypothesize that if the frequency of the recovery breath during hook breathing could be decreased there would be fewer opportunities for the rapid decrease in blood pressure. Breath hold or apneic training may facilitate a longer maintenance of the Valsalva maneuver with a concomitant decrease in the frequency of the recovery breath. Evidence from the extreme apneic capabilities of elite breath hold divers without prior oxygen supplementation (world records: male: 11 min: 35 sec, female: 7 min: 02 sec) and substantially longer durations following prior oxygen breathing (up to 24 min: 37 sec) (Elia et al., 2021b) suggest there is considerable room for reducing the frequency of recovery breaths and consequently improving the ability to maintain arterial hypertension during AGSM. This is evident in some experienced pilot trainers who demonstrated longer mean breathing cycle durations with a different muscle firing sequence (specifically the buccinator muscle) than a trainee group. They suggested that EMG feedback may be helpful in learning and enhancing AGSM performance (Chen et al., 2004). Further, the inability to hold one’s breath for greater than 20 seconds indicates diminished cardiac or pulmonary reserve and could serve as a screening indicator of reduced physiological capacity.

Breath holding (apnea) decreases arterial oxygen (oxyhemoglobin) saturation (Guimard et al., 2014; Bouten et al., 2022), and increases carbon dioxide concentrations (a primary stimulus for breathing) making prolonged Valsalva maneuver maintenance difficult (Bagavad et al., 2014). Acute muscular exercise also reduces breath hold time as metabolites such as H+ ions contributing to increased acidity also contribute to the impulse to breathe (Bagavad et al., 2014). For example, decreases in pre-frontal cortex deoxygenated hemoglobin were greater when breath holding was performed with cycling (ergometer) compared with rest (Allinger et al., 2025).

During apnea, heart rate and cardiac output are attenuated (Fico et al., 2022; Nobuhiro et al., 2024), reducing cardiac oxygen demand while peripheral oxygenation declines and cerebral oxygenation is relatively increased reflecting a redistribution of blood flow to prioritize cerebral perfusion (Costalat et al., 2013; Bouten et al., 2020). During apnea increased sympathetic activation, and norepinephrine release promote peripheral vasoconstriction, reducing blood flow to peripheral tissues and contributing to relative preservation of cerebral tissue oxygenation (Eichhorn et al., 2017). Apnea can induce rapid, active contraction of the spleen releasing more erythrocytes into the blood circulation. The slow recovery of splenic contractions may contribute to prolongation of successive, briefly repeated apnea attempts (Bakovic et al., 2003).

Single maximal duration breath holds in both trained and untrained divers did not elicit significant changes in electroencephalography (EEG), brain tissue oxygenation index, direct current potential, attention level, or processing speed immediately post-apnea (Ratmanova et al., 2016). However, responses can differ between trained and untrained breath hold divers. Both heart rate and cardiac output significantly decreased during a combination of apnea and 30 seconds of leg cycling exercise in experienced breath hold divers in contrast to a significant increase in the non-experienced breath hold group (Nobuhiro et al., 2024). Apnea-induced bradycardia is workload dependent with higher exercise intensity reducing the magnitude of heart rate slowing, likely reflecting heightened sympathetic nervous activity and an increased oxygen conserving effect (Wein et al., 2007) associated with the competing metabolic demands.

Although breath hold duration can be predicted by aerobic capacity, total lung volume, hematocrit and hemoglobin concentration, all of which are generally higher in males, breath hold duration did not differ between the sexes (Cherouveim et al., 2013). Breath hold duration is affected by the menstrual cycle, with the longest durations occurring during the mid-luteal phase possibly due to elevated estrogen and progesterone levels which influence both the ventilatory response and metabolic rate (Cherouveim et al., 2020).

In summary, although apnea decreases oxyhemoglobin saturation, increases carbon dioxide concentrations and induces hypertension, while exercise distribute metabolites contributing to increased acidosis, that heighten ventilatory drive, these effects are counterbalanced by reflex reductions in heart rate and cardiac output that contribute to oxygen conservation (Lindholm et al., 1999).

## Cardiovascular responses to high +Gz forces

### Cerebral blood flow

Following 20 minutes of centrifugation at 1.5 +Gz, cerebral blood flow velocity is reduced with no alteration in mean arterial pressure (Iwasaki et al., 2012; Konishi et al., 2019). In response to this low +Gz force, dynamic cerebral autoregulation is improved in a reflexive compensation for the reduction in steady-state global cerebral blood flow (Iwasaki et al., 2012). These cardiovascular responses can be seat position or anatomic height specific with no significant changes in mean arterial pressure at the heart level whereas at the middle cerebral artery level it was depressed (Konishi et al., 2018). Even when cardiac output is maintained, cerebral perfusion will diminish due to the increasing resistance of the cerebral flow circuit resulting from the +Gz-induced collapse of the extracranial veins. This response can start at moderate +Gz forces, becoming pronounced at approximately 4.5 +Gz (Cirovic et al., 2000). The limited role of elevated cardiac output may partly explain why aerobic training and high aerobic capacity do not confer +Gz tolerance benefits. Other factors such as stress, heat, and dehydration (starting at 3%) significantly reduce +Gz tolerance due to increased peripheral vasodilation and decreased plasma volume (Vashisth et al., 2017).

+Gz acceleration to approximately 6 +Gz without straining maneuvers reduces cerebral blood flow velocity to roughly half of normal values and can induce a caudal fluid shift of volume from both intra- and extracranial tissues (i.e. blood, lymphatic, cerebrospinal fluid), which intensifies with increasing +Gz (Kawai et al., 1997). Centrifuge runs at 2 to 5 +Gz (0.4-G/s onset rate) for 30 s reduced cerebral blood flow by 19 – 61% respectively, at the end of the onset and by 18 - 47%, respectively, in the last 20 s of the acceleration plateau. However, cerebral blood flow decreases less than cephalic arterial blood pressure, indicating that some mechanisms are activated to maintain cerebral perfusion under +Gz stress, such as a siphon effect and/or an autoregulatory compensation (Ossard et al., 1994).

Oxyhemoglobin and total hemoglobin were decreased during both short-term (5–20 s at 4-7 +Gz) and sustained (30 s at 7 +Gz) +Gz exposure. Contracting the rectus abdominus at higher intensities (6.2 to 36.8% of maximum voluntary isometric contraction (MVIC) force) was positively correlated with oxyhemoglobin changes during sustained 7 +Gz exposure helping to preserve brain blood volume. However, there was no significant correlation with vastus medialis contraction and cerebral oxyhemoglobin retention. Thus, even submaximal intensity abdominal muscles contractions provided some protection for brain blood flow (Kobayashi et al., 2002). Hence, employing more rapidly fatiguing maximal or near maximal isometric contractions may not provide additional benefits. The appropriate AGSM contraction intensity needs to be more fully investigated to optimally combine lower body and trunk vascular resistance, increased mean arterial pressures with enhanced muscle endurance.

### Cardiac responses (heart rate)

With high +Gz loads, heart rates (HR) can increase over 200 b.min^-1^ while left ventricular pressure may reach 300 mm Hg (Laughlin, 1986). There is an initial rapid rise in HR (<10 s) with increased +Gz that may mirror the initial baroreflex compensatory time frame. A HR response threshold or plateau that appeared after 7-seconds at 2.25 +Gz suggests that the vasoconstrictor response is more vital than cardiac output responses to high +Gz stresses (Goodman et al., 2000). Trainees with HR increases of less than 20% in the first 5 seconds had higher odds (adjusted Odds Ratio: 1.83), of 9 +Gz intolerance. Although lower +Gz tolerance was associated with smaller HR increases, this relationship appeared to be mitigated by improved AGSM effectiveness (adjusted Odds Ratio: 1.26). Tu et al. (2020b) suggested that low AGSM effectiveness and a small HR increase were separately associated with failure during high-+Gz exposure. While a smaller heart rate increase might initially be interpreted as superior aerobic conditioning reflecting superior cardiovascular reserve, HR responses during rapid onset +Gz stress also reflect baroreflex mediated compensations to reductions in carotid arterial pressure. High +Gz exposure has also been associated with electrocardiograph (ECG) rate and rhythm disturbances, observed in 73% of subjects, with atrial or ventricular ectopy in 67% of individuals. During G-LOC episodes, (average duration = 12.6 s), 33% of the individuals had atrial or ventricular ectopy (abnormal heart beats associated with arrhythmia). In addition, significant ventricular tachycardia (increased heart rate) and supraventricular tachycardia were observed during +Gz forces but not during the G-LOC period (Whinnery et al., 1990).

In summary, at higher +Gz forces, cerebral blood flow velocity is reduced, mean arterial pressure at the cerebral artery level is depressed, cerebral flow resistance is increased due to collapse of the extracranial veins, and there is evidence of decreased oxyhemoglobin and total hemoglobin. However, because the reduction in cerebral blood flow is less than the decrease cephalic arterial pressure, there are counterbalancing mechanisms, such as autoregulatory compensation, to maintain cerebral perfusion (Ossard et al., 1994). Furthermore, HR response thresholds suggest that reflexive vasoconstrictor mechanisms exert a greater influence on tolerance to high +Gz stresses than do cardiac output responses (Goodman et al., 2000). Accordingly, overemphasis on aerobic conditioning focused on improving maximal aerobic capacity may offer limited benefit for improving +Gz tolerance.

## Limb and trunk muscle responses to high +Gz forces

Long exposure to +Gz loads can impair +Gz tolerance. In one experiment, a 5-hr flight simulation diminished pilots’ functional state and high +Gz loads tolerance, increased the incidence and severity of visual disorders and cardiac arrhythmias, and elevated energy expenditure and cervical muscle fatigue (Khomenko et al., 2003). Bain et al. (1995) reported that knee extensor forces generated during Simulated Air Combat Maneuvers (SACM) at 7 +Gz approximated 35% of MVIC force and did not change significantly during the SACM. Contrary to the authors’ expectations, the inability to continue performing effective AGSM cannot be reliably ascribed to central fatigue or fatigue of large muscle groups tested. Another similar study by Bain et al. (1994) found that during 7 +Gz plateaux, electromyographic (EMG) activity of various upper and lower body muscles ranged from 26-48% of MVIC and relative force outputs ranged from 30-61% of MVIC. With a rapid onset to 6 +Gz, the mean EMG root mean square amplitude of lower extremity muscles (biceps femoris, vastus lateralis, lateral gastrocnemius) decreased 61.45%, trunk muscles (erector spinae, external obliques) reduced 3.45% during the AGSM, while EMG mean power frequency did not significantly change. They then divided their group of 10 participants into “good” and “poor” +Gz tolerance durations (mean 175.6 s and 41.7 s, respectively) and found that the good group exhibited a leveling of EMG activity after the first 25% of the centrifuge exposure, contrasting with the poor group who showed a continual decrease, particularly in the lower extremity muscles (Cornwall and Krock, 1992). Neck and shoulder muscles activity increased with higher +Gz forces, however inexperienced pilots had significantly higher muscle activity than experienced pilots and thus previous high +Gz exposure may result in lower muscle activation during SACM (Honkanen et al., 2017). Modeling of the lumbar spine indicates that activation of lumbar muscles provides protection for maintaining the lumbar spine and reduced spinal injury risk to less than 10% probability under +Gz stress (Lei et al., 2024). However, Honkanen et al. (2018) observed no association between approximately 5 years of flight experience and increased measures of cervical or lumbar spinal degeneration. Anticipatory pre-tensing of leg and abdominal muscles prevented visual greyout completely during simulated space flight launches, and decreased greyout occurrence from ~80% frequency to ~37% with simulated re-entry (Pollock et al., 2024). Central light loss after anti-G trouser deflation during 4-7 +Gz exposure was only modestly improved (approximately 0.5 +Gz) by muscle tensing (Stevenson and Scott, 2014). In addition, lumbar supports of 7-, 14-, and 26-mm increased average EMG 12% - 14% and EMG power spectrum area 20% - 44% compared to no additional lumbar support (Oksa et al., 2003).

Hence, the scant evidence suggests that isometric contractions during AGSM are typically of moderate intensity, with the activation levels decreasing over longer durations of AGSM. While alterations in muscle contraction intensity may impair the ability to maintain hypertension and blood volume in the upper body, pilot ability to monitor changes in muscle activation may be beneficial for maintaining sufficient levels of intensity during prolonged AGSM. Onboard data acquisition systems can provide physiologically useful information (Newman and Callister, 1999). EMG biofeedback with 58 Brazilian Air Force cadets facilitated motor learning of AGSM by selectively improving activation in targeted muscles (gastrocnemius, vastus medialis, and rectus abdominis), however, effects varied by muscle group, suggesting the need for tailored instructional strategies (Massaferri et al., 2025). Not only can EMG feedback illustrate the changes in the intensity of lower body and trunk muscle contractions, but an algorithm that monitors the degree and decay of the gastrocnemius EMG signal can generate warning signals regarding the probability of G-LOC occurrence (Kim et al., 2017). Choi et al. (2015) also found a rapid decay in muscle activation (EMG), three seconds before G-LOC, compared to a normal +Gz force withstanding phase and thus developed an algorithm which can detect and warn regarding G-LOC prognosis. Thus, surface EMG monitoring is considered as an affordable and useful tool providing real-time feedback to improve pilot’s muscle control with AGSM (Pires Junior et al., 2024).

## Possible conditioning strategies

### Nutritional strategies

Some nutritional strategies may be able to increase the duration of the Valsalva maneuver and reduce the recovery breaths (hook breathing). Lower O_2_ levels can be achieved at breath hold breakpoint with fasting (Lindholm et al., 2007). Overnight fasting was implemented to decrease metabolism and thus minimize oxygen consumption resulting in a 22% improvement in apneic duration (Schagatay and Lodin-Sundstrom, 2014). Acute dietary nitrate delivered by ingesting beetroot juice 2.5 hours prior to testing increased apneic duration by decreasing metabolic costs (Engan et al., 2012) and increasing arterial oxygen saturation (Patrician and Schagatay, 2017). While fasting or other nutritional protocols may not be pragmatic for pilots, the underlying mechanisms could provide the basis for useful supplementation to improve +Gz tolerance.

### Apneic and hypoventilation training

Since AGSM involves not only hook breathing but also high-intensity isometric contractions of the lower body and trunk, it may be considered a predominantly anaerobic, fatiguing activity whose endurance may be amenable to improvement through targeted training. In this context, hypoventilation and apnea-based training paradigms have been proposed as potential strategies to enhance anaerobic performance and fatigue resistance relevant to AGSM execution.

### Anaerobic training

A meta-analysis by Boulares et al. (2025) reported hypoxic sprint training induced by voluntary hypoventilation improved sprint-fatigue resistance and glycolytic capacity as well as enhancing muscle strength and hypertrophy. Similarly, a perspective review reported that 77% of the reported studies with hypoxic repeated-sprint training found it to be an efficient anaerobic training intervention (Faiss et al., 2025). Proposed underlying mechanisms included enhanced microcirculatory vasodilation leading to improved muscle perfusion and/or oxygenation (Delahoche et al., 2005), increased muscular phosphocreatine availability (Faiss et al., 2025) and a greater reliance on glycolytic metabolism (Woorons et al., 2019). Reduced tissue oxygenation and elevated lactate concentrations [La] during voluntary hypoventilation may lead to greater tolerance of intramuscular acidic environments (Woorons et al., 2010).

### Apnea training

In line with these findings, a meta-analysis by de Asis-Fernadez et al (de Asis-Fernandez et al., 2022). indicated that apnea training can be used as a strategy to enhance anaerobic performance associated with increased maximum blood lactate concentrations, although no evidence was found for improvements in aerobic measures. Consistent with this scope of adaptation, breath-hold divers capable of sustaining prolonged apnea demonstrate reduced blood acidosis, diminished oxidative stress, and lower basal metabolic rates, alongside increases in hematocrit, erythropoietin concentration, hemoglobin mass and lung volumes which may contribute to improved performance (Lemaitre et al., 2010).

Beyond performance analogs, several studies have directly examined the effects of apnea training on breath-holding capacity and autonomic responses. Following 13 days of apnea training consisting of five submaximal apneas and one maximal duration apnea per day, both total apnea duration (pre-test: 44s vs post-training: 75s) and struggle phase (involuntary breathing movements) duration (18s vs. 45s) increased substantially. Training was also associated with accentuated bradycardia at rest, and during a cognitive stressor (Stroop test) but not during an exercise-based stressor (handgrip), indicating context-specific autonomic adaptations rather than generalized exercise transfer (O'Croinin et al., 2025). Similarly, two weeks of apneic training (five maximal duration breath holds per day) increased apneic duration by 33% which was accompanied by increases in erythrocyte concentration, hemoglobin saturation, and peak mean arterial pressure, with the latter suggested to be associated with the greater breath hold time (Bourdas and Geladas, 2024).

Longer-term training studies further support the specificity of apnea-based adaptations. Groups exposed to two months of either apnea-specific training, or combined strength, endurance, mobility, and cardiovascular training both demonstrated greater apneic duration, however improvements were significantly greater following apnea-specific training versus physical interval training alone (Schagatay et al., 2000). In addition, three months of an apnea training intervention consisting of repeated 20 second intervals followed by 40 second recoveries during low intensity cycling (30% VO_2_ maximum for 1 hour); resulted in significant increases in static apnea duration, accompanied by accentuated bradycardia (lower heart rates), post-apnea decreases in venous blood pH, increased lactic acid concentration, and attenuation of post-apnea oxidative stress. During dynamic apnea performed with dynamic forearm exercise, blood acidosis and oxidative stress were similarly decreased, indicating breath-hold (apnea) training improves tolerance to hypoxemia (Joulia et al., 2003). The interaction between apnea and the intensity of concurrent exercise appears to be a critical determinant of physiological responses. During low-intensity exercise, apnea-induced bradycardia and peripheral vasoconstriction are preserved; however, short duration, high-intensity exercise induces tachycardia and peripheral vasodilation due to increased sympathetic activation and parasympathetic withdrawal. Nevertheless, exercise can still be sustained during breath holding since energy demands increasingly rely on intrinsic O_2_ stores and anaerobic metabolism (Walsh and G.R., 2025; Walsh et al., 2025). Consistent with this, breath hold training has been shown to improve respiratory endurance (Bagavad et al., 2014), and combining breath holding with low intensity aerobic training which induces moderate hypoxia, can be used to increase lactate concentrations [La] and tolerance that would typically be observed during high-intensity aerobic training (Guimard et al., 2021). Moreover, the combination of anaerobic exercise, such as repeated sprint training, with voluntary hypoventilation has been demonstrated to increase the number of sprint repetitions achieved (Ait Ali Braham et al., 2024). A meta-analysis by Massini et al. (2023) further reported apnea durations were similarly improved by breath-hold training, muscle strength and cardiorespiratory training (physical) or cross-training combining breath hold exercises with physical training.

With respect to chemosensory and hematological adaptations, chronic exposure to elevated carbon dioxide (CO_2_) would be expected to reduce ventilatory sensitivity to CO_2_ by way of the hypercapnic ventilatory response (Bourdas et al., 2015). However, CO_2_ sensitivity was not diminished either acutely (after 5 maximal breath holds) or chronically (after 14 days of breath hold training: 5 maximal breath holds per day) (Bourdas et al., 2015). Likewise, although acute apnea induces splenic contraction and transient release of erythrocytes into the circulation, repeated apnea training does not appear to augment this response. Specifically, training durations ranging from two weeks (10 maximal apneas per day) (Engan et al., 2013), to 6 weeks (4 apneas per day; 5 static apneas per day) (Elia et al., 2021a; Bouten et al., 2022), nor 8 weeks of apnea training (5 static apneas per day; 5 times weekly) (Bouten et al., 2019; Yang et al., 2022) did not result in sustained increases in splenic contraction, circulating erythrocytes, or hemoglobin concentration. Bouten’s group suggest that longer and/or more intense training sessions are probably necessary to induce changes in hematological parameters (Bouten et al., 2022; Bouten et al., 2025).

### Hypoventilation training

While numerous studies have shown hypoventilation-induced enhancements of anaerobic performance, including reduced repeated sprint fatigue, and changes in neuromuscular activity (Trincat et al., 2017; Fornasier-Santos et al., 2018; Lapointe et al., 2020; Woorons et al., 2024; Lorinczi et al., 2025; Precart et al., 2025; Raberin et al., 2025), not all investigations have demonstrated consistent positive effects (Woorons et al., 2008; Rosa et al., 2024). For example, three weeks of sprint interval training with voluntary hypoventilation improved aerobic capacity and oxidative metabolism, but did not enhance anaerobic metabolic capacity within this relatively short training period (Chiba and Yasuda, 2026). Moreover, while voluntary hypoventilation does not necessitate exposure to high altitude or artificial hypoxic environments, and may therefore represent an accessible training modality, training in hypoxic conditions (i.e., hypoventilation) may decrease training loads (Devantay et al., 2025).

In summary, hypoventilation training, either alone or in combination with anaerobic based exercise (e.g., sprint training), can induce many adaptations beneficial to improving apnea performance, including augmentation of anaerobic and glycolytic capacities, increased tissue oxygenation, tolerance to acidosis, erythropoietin and hemoglobin concentrations, respiratory muscle endurance and context-specific autonomic adaptations. Given that +Gz duration tolerance is limited, in part by the onset of fatigue (Burton, 1999), these adaptations may support improved AGSM performance by enabling prolonged Valsalva maneuvers, reduced recovery breath frequency, and greater endurance of trunk and lower-body isometric muscle contraction.

### Respiratory muscle fatigue training

During a maximal duration breath hold, involuntary respiratory muscle contractions against a closed glottis (i.e., involuntary breathing movements associated with the struggle phase) occur, contributing to respiratory fatigue even without voluntary muscle contractions (Hubbard et al., 2025). In the same study, decrements in diaphragmatic strength and overall inspiratory and expiratory muscle strength were observed in a subset of participants, suggesting that limitations in oxygen availability may contribute to respiratory muscle fatigue during breath holding. Furthermore, inhalation flow diminishes with increasing +Gz forces due to breathing difficulty associated with the +Gz load and anti-G suit inflation, whereas exhalatory mechanics were not affected (Whitley, 1997). This would impact the effectiveness of the recovery breath during the AGSM. Average SACM durations of 358 ± 15 s induced respiratory muscle fatigue with increased inspiratory work, as evidenced by lower pressure generation during a maximal AGSM and decreased EMG frequency shifts (i.e., diaphragm, scalenes, intercostals) (Bain et al., 1997).

A single session of deep abdominal muscle strengthening exercises with 10 seconds of breath holding at the end of each repetition (5 sets of 10 repetitions) improved respiratory function (transversus abdominus contractility, forced vital capacity and expiratory ventilation) and lumbar stabilization (Kim and H., 2013). Four weeks of inspiratory muscle training (20 minutes per day, 3 days per week) decreased peripheral oxygen saturation recovery times by more than three seconds and increased forced expiratory volume and maximum inspiratory pressure by a large magnitude compared to high intensity interval training (de Asis-Fernandez et al., 2020). Another study found that while six weeks of respiratory muscle training significantly improved respiratory muscle strength, it had limited benefit for improving AGSM-induced fatigue (Yang et al., 2007). Inspiratory muscle strength and endurance were enhanced following 10 weeks of inspiratory muscle training in well-trained athletes, however, there was no augmentation of aerobic capacity or arterial O_2_ desaturation during maximal graded exercise (Inbar et al., 2000). Accordingly, respiratory muscle training may mitigate fatigue associated with apnea-induced involuntary respiratory muscle contractions, while also improving inspiratory and expiratory performance under the increased mechanical loads imposed by anti-G suits +Gz-related thoracic compression.

### Physical training (aerobic, anaerobic, muscle strength and endurance)

The ability to withstand high +Gz stress requires rapid cardiovascular responses (Forster and Whinnery, 1990). Although aerobic training can enhance parasympathetic tone, resulting in a reduced heart rate during +Gz stress, it does not alter heart rate responsiveness to higher +Gz stress (Forster and Whinnery, 1990) nor enhance +Gz tolerance (Ludwig et al., 1987; Whinnery and Parnell, 1987). Royal Australian air force fighter pilots perceived physical fitness to be important and had higher rates of participation in aerobic than anaerobic activities. However, it was determined that good but not exceptionally high levels of aerobic power were unlikely to influence +Gz tolerance (Newman et al., 1999). According to Kolegard et al. (2013), relaxed +Gz-tolerance is unaffected by physical training habits, however, the exercise pressor response was reduced with endurance training compared to an enhancement with strength training. Tu et al. (2020b) recommended fighter pilots’ physical training for +Gz tolerance should include both strength and aerobic training since moderate volumes of aerobic training would strengthen cardiovascular function and reduce recovery duration from repetitive +Gz exposures. In a sample of cadets from the Republic of Korea air force academy, those who successfully completed the G-LOC test demonstrated faster 3-km run times and higher overall physical activity levels than those who failed the test (Sung et al., 2023; Sung and Lee, 2024). On the other hand, inactivity (14 days of bed rest) can reduce +Gz tolerance by 67% (Newsom et al., 1977).

Increases in systemic blood pressure are directly related to skeletal muscle force production (Lind and McNicol, 1967). However, while strength-trained individuals exhibited higher mean arterial pressures during a 40 second isometric contraction at 50% of MVIC than endurance trained or untrained individuals, there were no significant differences in +Gz tolerance suggesting +Gz tolerance may not be significantly affected by the dynamic (isotonic) muscle strength or endurance training used in these studies (Kolegard et al., 2013; Park et al., 2016). Slungaard et al. (2019) reported that a 12-week aircrew conditioning program showed a trend toward improve straining +Gz tolerance, possibly mediated by enhanced systemic vascular resistance. Although the program did not significantly increase overall +Gz endurance, a greater proportion of participants completed the SACM profiles, due to a reduction of the relative physical workload required at a given +Gz level (Slungaard et al., 2019).

In summary, despite decades of operational experience and research on +Gz exposure and tolerance, there is a scarcity of systemic training studies examining the independent and combined effects of aerobic, anaerobic, muscle strength, or muscle endurance training on +Gz tolerance. The limited training studies show mixed and sometimes conflicting results. From a physiological standpoint, enhanced muscular endurance would be expected to support prolonged execution of AGSM, but empirical evidence remains sparse. Whether increases in muscle strength and mass might further contribute by augmenting peripheral arterial resistance and limiting the cephalocaudal fluid shift, blood displacement remains insufficiently explored. Applying the principles of training specificity, the isometric contractions central to AGSM performance are primarily anaerobic in nature, suggesting that targeted anaerobic or isometric training, in isolation or combination might prove beneficial. Finally, because pilots must maintain coordinated motor control and make rapid, complex decisions under high +Gz stress, training approaches that integrate physical and cognitive demands are likely to be particularly relevant.

## Conclusions

The ability to tolerate high +Gz forces is essential for aircraft performance capabilities, pilot health, and the protection of life. While technologies such as anti-G suits, pressurized breathing, seat configurations, audio-visual displays to monitor physiological responses, and other techniques aid in augmenting +Gz tolerance, AGSM also plays a pivotal role. While centrifuge conditioning is crucial for building +Gz tolerance, there are few centrifuges available and exposure time is constrained. Improving AGSM techniques should increase arterial resistance to cephalocaudal blood movement helping to maintain cerebral blood flow and volume and prevent hypoxemia leading to vision difficulties, A-LOC and G-LOC. One of the weaknesses of hook breathing during AGSM is the recovery breath both in terms of maintaining cerebral blood oxygen levels (expiration and inspiration difficulties) but also preventing a dramatic decrease in cephalic mean arterial pressure. Hence, one of the highest “Return on Investment” knowledge gaps would be to adopt some breath hold (apneic) training techniques to prolong the Valsalva maneuver and decrease the frequency of recovery breaths. Furthermore, as lower body and trunk isometric contractions are susceptible to fatigue effects, they may benefit from more AGSM task specific isometric muscle endurance and possibly strength training. Implementing, practising, and training for rapid rate of isometric muscle contraction onset (ballistic-intent contractions) (Behm et al., 2025) may provide pilots with an advantage in rapidly initiating AGSM countermeasures. In addition, anaerobic training may increase glycolytic capabilities and acidic tolerance to further promote endurance. Nutritional or supplemental strategies such as the ingestion of sodium bicarbonate to buffer acidosis shows mixed results in the literature (Williams et al., 2026). As the AGSM is not performed in isolation when operating the aircraft, integrated physical and cognitive training should be a hallmark of successful high +Gz tolerance training as well. Furthermore, another high “Return on Investment” knowledge gap would be related to the limited time available for muscle strength, endurance and cognitive training for pilots, and thus minimal weekly frequencies and volumes of such training must be established (Behm et al., 2024). As there is comparably little training specific research on these topics after all these years of +Gz tolerance experience, it is still an open area for more applied literature (see **Table 1** for summary of knowledge gaps and potential future research.

**Table 1:** Anti- G straining maneuvers (AGSM) knowledge gaps and future research possibilities.

Acute responses
What intensity of lower body and trunk isometric contractions are necessary to maintain cerebral blood flow under high Gz force environments? Are there specific anthropometric (e.g., muscle and fat mass, body mass index, height, sex, age) and physiological characteristics (e.g., neuromuscular: grip strength, leg strength, and endurance, and cardiorespiratory: breath hold duration, forced expiratory volume, total lung volume) that enhance AGSM performance (i.e., maintenance of cerebral blood flow)? Is there an association/correlation and if there is an association, is it greater between muscle strength or muscle endurance with AGSM efficiency? What is the extent of change in cardiovascular variables (i.e., blood pressure, cerebral blood flow, heart rate and heart rate variability) between the Valsalva maneuver, recovery breath (most susceptible period for G-LOC, vision and performance deficits) and isometric contractions? Which Valsalva durations and isometric contraction intensities are most effective at maintaining cerebral blood flow when subjected to high Gz forces? Do menstrual cycle phases have differential effects on AGSM?
Chronic training
Can the current recommendation of 3-second Valsalva followed by a recovery breath (dramatic blood pressure reduction and impaired cerebral blood flow is the most susceptible period for G-LOC, vision or performance impairments) be prolonged with practice and breath hold training? Is breath hold (apnea or hypoventilation) training effective for increasing Valsalva maneuver durations? Is AGSM enhanced with breath hold training alone or can there be additive effects (more effective)? when incorporated with Valsalva maneuvers and isometric contractions (task and training specificity)? Should these three variables be trained concurrently or consecutively? Is muscle strength or endurance training, anaerobic or aerobic training or a combination more effective for improving AGSM? Can respiratory muscle training enhance Valsalva maneuver durations and reduce the frequency of recovery breaths (mitigate fatigue associated with apnea-induced involuntary respiratory muscle contractions, while also improving inspiratory and expiratory performance under the increased mechanical loads imposed by anti-G suits +Gz-related thoracic compression)? Is AGSM task specific isometric resistance training more effective for AGSM efficiency than dynamic resistance training (e.g., free weights, machines, resistance bands) or would a combination have greater effectiveness? Can ballistic-intent resistance training enhance and accelerate the blood pressure increase with AGSM? Would an integration of physical (e.g., Valsalvas and muscle strength/endurance training) and cognitive (cockpit operational responses) training provide a more effective training environment? Can lower body negative pressure or seated inversion to upright position training provide similar G tolerance training adaptations as centrifuge training? As there are time constraints for pilots, what are the minimal weekly frequencies, training durations (i.e., weeks or months) and volumes (i.e. sets, repetitions) for AGSM (e.g., Valsalva maneuver, muscle strength and endurance) training? Are there nutritional or supplemental (e.g., sodium bicarbonate, caffeine, beet root juice, fasting, or others) strategies to enhance AGSM (e.g., decrease muscle and blood acidosis with prolonged or repetitive Valsalva maneuvers and isometric contractions)?
